# Stress-Activated Protein Kinases in Human Fungal Pathogens

**DOI:** 10.3389/fcimb.2019.00261

**Published:** 2019-07-17

**Authors:** Alison M. Day, Janet Quinn

**Affiliations:** Faculty of Medicine, Institute for Cell and Molecular Biosciences, Newcastle University, Newcastle Upon Tyne, United Kingdom

**Keywords:** Hog1, *Cryptococcus*, *Candida albicans*, *Aspergillus fumigatus*, SAPK, stress signaling, fungal pathogenesis

## Abstract

The ability of fungal pathogens to survive hostile environments within the host depends on rapid and robust stress responses. Stress-activated protein kinase (SAPK) pathways are conserved MAPK signaling modules that promote stress adaptation in all eukaryotic cells, including pathogenic fungi. Activation of the SAPK occurs via the dual phosphorylation of conserved threonine and tyrosine residues within a TGY motif located in the catalytic domain. This induces the activation and nuclear accumulation of the kinase and the phosphorylation of diverse substrates, thus eliciting appropriate cellular responses. The Hog1 SAPK has been extensively characterized in the model yeast *Saccharomyces cerevisiae*. Here, we use this a platform from which to compare SAPK signaling mechanisms in three major fungal pathogens of humans, *Candida albicans, Aspergillus fumigatus*, and *Cryptococcus neoformans*. Despite the conservation of SAPK pathways within these pathogenic fungi, evidence is emerging that their role and regulation has significantly diverged. However, consistent with stress adaptation being a common virulence trait, SAPK pathways are important pathogenicity determinants in all these major human pathogens. Thus, the development of drugs which target fungal SAPKs has the exciting potential to generate broad-acting antifungal treatments.

## Introduction

Stress responses are essential for pathogenic fungi to survive hostile environments encountered in the host (Brown et al., 2017), and it is apparent that stress-activated protein kinase (SAPK) pathways are central in mediating such responses and virulence in many fungal pathogens. SAPKs were first identified in the model yeast *Saccharomyces cerevisiae* where the High Osmolarity Glycerol (HOG) pathway was shown to regulate the cellular response to osmotic stress (Brewster et al., 1993). Subsequently, mammalian homologs, of the yeast Hog1 SAPK, namely p38 and JNK (c-Jun N-terminal kinase), were discovered (Galcheva-Gargova et al., 1994; Han et al., 1994). The high level of conservation between mammalian and yeast SAPKs is demonstrated by the fact that human p38 can complement the stress-sensitive phenotypes of the *S. cerevisiae hog1*Δ mutant (Han et al., 1994). It is now recognized that SAPKs are found in all eukaryotic cells, and are among the most evolutionarily conserved stress-signaling proteins (Nikolaou et al., 2009). There are three tiers of protein kinases within each SAPK module, comprising of a MAPKKK which phosphorylates and activates a MAPKK, which in turn phosphorylates and activates the terminal SAPK. Once active, the SAPK phosphorylates a range of different nuclear and cytoplasmic substrates thus triggering a myriad of distinct cellular responses.

The HOG pathway in *S. cerevisiae* is arguably the best characterized SAPK pathway, and using this as a reference we describe related SAPK pathways in three major fungal pathogens of humans; *Candida albicans, Aspergillus fumigatus*, and *Cryptococcus neoformans*.

## *Saccharomyces Cerevisiae* Hog1p

Here, the role and regulation of Hog1p in response to osmotic stress will be summarized as, mechanistically, this is the best characterized system. However, it should be noted that *S. cerevisiae* Hog1p is also implicated in other stress responses including oxidative stress (Bilsland et al., 2004), cold stress (Hayashi and Maeda, 2006), hypoxia (Hickman et al., 2011), the toxic metabolite methylglyoxyl (Aguilera et al., 2005), arsenite (Lee and Levin, 2018), weak acid stress (Mollapour and Piper, 2007), and heat stress (Winkler et al., 2002).

**Figure 1:**
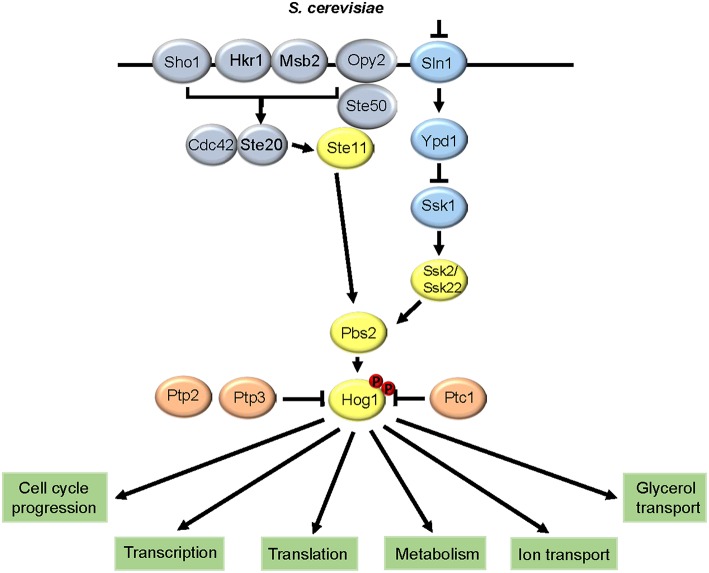
HOG pathway architecture in *Saccharomyces cerevisiae*. The two signaling branches that converge to regulate Hog1p, and the major downstream responses, are shown.

The signaling pathways that regulate the relay of osmotic stress signals to Hog1p in *S. cerevisiae* is summarized in Figure 1. Within the three-tiered SAPK module, Hog1p is regulated by three MAPKKKs, the Ssk2p/22p orthologs and Stellp, and a single MAPKK Pbs2p (Posas and Saito, 1997). These upstream kinases regulate phosphorylation of Hog1p on threonine-174 and tyrosine-176 within the conserved TGY motif, which activates and induces the nuclear accumulation of the kinase (Ferrigno et al., 1998). Osmotic stress-sensing and signaling to the Hog1p SAPK module has been extensively characterized and involves two pathways that function redundantly (for excellent reviews see Saito and Posas, 2012; Brewster and Gustin, 2014). These pathways comprise the Sln1p two-component related signaling pathway, and the Sho1p pathway, which converge at the level of the Pbs2p MAPKK. Relevant for this review, it appears that whilst two-component related pathways are widely used to modulate SAPK activation in pathogenic fungi, Sho1-signaling to SAPKs is not universally conserved. Two-component phosphorelay pathways are widely used in bacteria to respond to environmental signals (Egger et al., 1997), but fungi have adapted a more complex three tier phosphorelay system. In *S. cerevisiae*, this comprises of the Sln1p histidine kinase, the Ypd1p phosphorelay protein, and the Ssk1p response regulator. Osmotic stress causes a loss of turgor pressure in the membrane, which inactivates the Sln1p histidine kinase and thus phosphorylation of Ssk1p via Ypd1p is inhibited (Posas et al., 1996). Dephosphorylated Ssk1p is a potent activator of the Ssk2p/22p MAPKKKs (Posas and Saito, 1998). Notably, loss of Sln1p or Ypd1p results in a lethal phenotype in *S. cerevisiae* due to accumulation of unphosphorylated Ssk1p and the consequent hyperactivation of Hog1p (Maeda et al., 1994). In the Sho1p branch, the Stellp MAPKKK phosphorylates Pbs2p (Tatebayashi et al., 2006), and Pbs2p acts as a scaffold to allow interaction between itself, Sho1p, Ste11p, and Hog1p (Posas and Saito, 1997). Many proteins have been implicated in the transmission of the osmosignal from Sho1p to Ste11p-Pbs2p (Tatebayashi et al., 2006), and there has been some debate regarding the osmosensor of the Sho1p pathway with two membrane-located mucins, Msb2p and Hkr1p, suggested as potential candidates (Tanaka et al., 2014). However, it has recently been shown that Sho1p is the osmosensor, with osmotic stress-induced structural changes in the transmembrane domains of Sho1p triggering binding to the cytoplasmic adaptor Ste50p, resulting in Hog1p activation (Tatebayashi et al., 2015). In addition to the two upstream branches promoting Hog1p phosphorylation, this kinase is also subjected to negative regulation via phosphatase action with the major phosphatases comprising of the phosphotyrosine phosphatases Ptp2p and Ptp3p (Wurgler-Murphy et al., 1997) and the 2C Ser/Thr phosphatase Ptc1p (Warmka et al., 2001).

Following activation, Hog1p mounts a multi-layered response to osmotic stress via regulating both nuclear and cytoplasmic targets (Saito and Posas, 2012; Brewster and Gustin, 2014). Nuclear functions of Hog1p include the regulation of stress-protective gene expression, including genes involved in glycerol production and ion transport, via the Msn2p/4p, Smp1p and Hot1p transcriptional activators, and the Sko1p repressor (de Nadal and Posas, 2010). Of these perhaps the best characterized is the Sko1p ATF/CREB repressor which is a substrate of Hog1p and is phosphorylated following osmotic stress. This converts the Sko1p-Cyc8p-Tup1p complex from a repressor to an activator resulting in the recruitment of the SWI/SNF and SAGA chromatin-modifying complexes which induce binding of RNA polymerase II and transcription of osmotic stress genes (Proft and Struhl, 2002). In addition, Hog1p regulates delays in the cell cycle at G1, S, and G2 phases to permit stress adaptation prior to cell-cycle progression (Clotet and Posas, 2007). Cytoplasmic functions of Hog1p include the regulation of metabolic enzymes (Dihazi et al., 2004), and the closing of the Fps1p aquaglyceroporin (Lee et al., 2013). Interestingly, although nuclear accumulation of Hog1p is required for transcription of osmotic stress genes, preventing nuclear accumulation does not impact osmotic stress resistance (Westfall et al., 2008). This suggests that cytoplasmic functions of Hog1p, rather than transcriptional responses in the nucleus, are more important in promoting osmotic stress resistance in *S. cerevisiae*.

## *Candida albicans* Hog1

**Figure 2:**
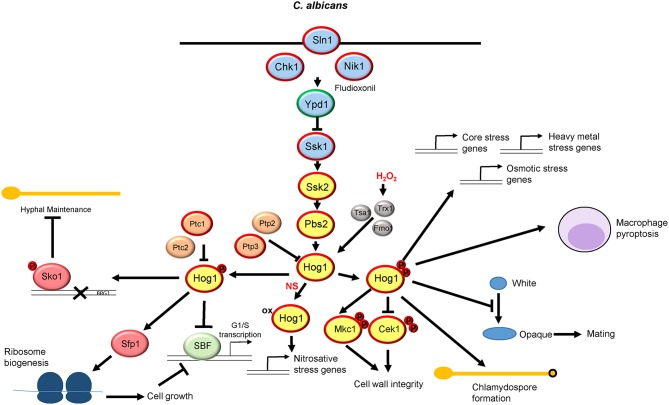
The Hog1 SAPK pathway in *Candida albicans*. Both the signaling proteins that regulate Hog1 and the downstream responses are shown. See text for details. Those proteins circled in red are required for *C. albicans* virulence, whereas Ypd1 circled in green promotes virulence upon inactivation.

A number of *Candida* spp. are pathogens of humans with *C. albicans* being the most important causing 400,000 life threatening systemic infections per annum (Brown et al., 2012). The role and regulation of the Hog1 pathway in *C. albicans* is summarized in Figure 2. *HOG1* was first identified in *C. albicans* in 1996 as a gene that could rescue the osmotic stress sensitive phenotype of the *S. cerevisiae hog1*Δ mutant (San Jose et al., 1996). Subsequently, *C. albicans* Hog1 has been found to be activated and/or promote resistance to diverse stress conditions likely to be encountered in the host or during antimicrobial therapy. For example, Hog1 is important for cellular responses to osmotic and oxidative stresses (Alonso-Monge et al., 2003; Smith et al., 2004), nitrosative stress (Herrero-de-Dios et al., 2018), metal availability (Kaba et al., 2013), various drugs (Smith et al., 2004; Kelly et al., 2009), antimicrobial peptides (Vylkova et al., 2007; Argimon et al., 2011; Hayes et al., 2013), increased glucose levels (Rodaki et al., 2009), and photodynamic inactivation (Chien et al., 2018). Whereas *hog1*Δ cells generally display impaired resistance to the aforementioned stresses, loss of Hog1 increases resistance to cell wall damaging agents such as calcofluor white and congo red (Alonso-Monge et al., 1999). This is due to inappropriate activation of the Cek1 MAPK pathway in *hog1*Δ cells via the cross talk phenomenon (Eisman et al., 2006).

In addition to stress resistance, Hog1 is also implicated in a multitude of cellular processes in *C. albicans* including chlamydospore production (Alonso-Monge et al., 2003) and morphogenetic switching (Alonso-Monge et al., 1999; Enjalbert et al., 2006; Su et al., 2013), with *hog1*Δ cells displaying filamentous growth in the absence of any morphogenetic cues (Enjalbert et al., 2006). Hog1 also impacts on respiratory metabolism (Alonso-Monge et al., 2009), cell cycle regulation (Correia et al., 2016; Sellam et al., 2019), and white-opaque switching and mating (Liang et al., 2014). Hog1 function is also intimately connected in regulating processes triggered following Candida-phagocyte interactions including induction of macrophage pyroptosis (O'Meara et al., 2018), and the dynamic reorganization of the fungal cell wall initiated in response to neutrophil extracellular trap (NET)-mediated damage (Hopke et al., 2016), the latter of which may be linked to the deregulation of the Cek1 MAPK in *hog1*Δ cells (Eisman et al., 2006).

As perhaps expected due to the involvement of Hog1 in multiple processes in *C. albicans*, this SAPK is essential for virulence. This was first shown in 1999 when, in a murine systemic model of candidiasis, mice injected with *hog1*Δ cells were found to survive significantly better than those injected with wild type cells (Alonso-Monge et al., 1999). Subsequently, Hog1 has also been found to be important for fungal survival following phagocytosis (Arana et al., 2007), and in promoting colonization of the mouse gastrointestinal tract (Prieto et al., 2014). As Hog1 regulates the yeast to hyphal switch, which is important for *C. albicans* pathogenesis (Lo et al., 1997), it is possible that this underlies Hog1-mediated virulence. However, a Hog1-pathway mutant that showed impaired stress resistance but no defect in morphological switching displayed reduced virulence analogous to *hog1*Δ cells suggesting that Hog1 contributes to virulence independently of its role in morphological switching (Cheetham et al., 2011).

Within the *C. albicans* SAPK module, Hog1 is activated via the Pbs2 MAPKK and the Ssk2 MAPKKK. Thus, in response to many of the stresses outlined above, Hog1 becomes phosphorylated on the conserved TGY motif in a Pbs2 and Ssk2-dependent manner. Consequently *C. albicans* cells lacking *PBS2* or *SSK2* largely phenocopy *hog1*Δ cells (Arana et al., 2005; Cheetham et al., 2007, 2011). Interestingly, homology between *C. albicans* Pbs2 and *S. cerevisiae* Pbs2p appears to be restricted to the C-terminus, with the N-terminal region of *C. albicans* Pbs2 being considerably shorter than that of the *S. cerevisiae* protein (Cheetham et al., 2007). However, the activating phosphorylation sites are conserved and mutation to non-phosphorylatable alanine residues generates identical phenotypes to those displayed by *pbs2*Δ cells (Cheetham et al., 2011). In contrast to *S. cerevisiae*, Pbs2 is activated by a sole MAPKKK Ssk2 in *C. albicans* (Cheetham et al., 2007). Although homologs of the Sho1 osmosensing branch are conserved in this fungal pathogen, neither Sho1 nor the downstream Ste11 MAPKKK function to relay osmotic stress signals to Hog1 (Roman et al., 2005; Cheetham et al., 2007). It was thought that this may be due to the truncated N-terminal region of *C. albicans* Pbs2 (Cheetham et al., 2007) which lacks the Sho1-binding domain present in *S. cerevisiae* Pbs2p (Tatebayashi et al., 2003). However, a chimeric protein comprising the N-terminus of *S. cerevisiae* Pbs2p fused to the C-terminus of *C. albicans* Pbs2, whilst functional, was unable to activate Hog1 in response to osmotic stress in the absence of Ssk2 (Cheetham et al., 2011). Collectively, these findings illustrate that the Sho1-Ste11 pathway in *C. albicans* does not respond to changes in osmolarity.

As in *S. cerevisiae*, the *C. albicans* Hog1 module is regulated by a two-component related phosphorelay system (Smith et al., 2010). However, in contrast to *S. cerevisiae, C. albicans* has three sensor histidine kinases, Sln1, Nik1, and Chk1 although their mechanism of action is unclear. *C. albicans* Sln1 is homologous to *S. cerevisiae* Sln1p and contains two transmembrane domains indicating a conserved plasma membrane location (Salas-Delgado et al., 2017). *C. albicans* Sln1 is able to rescue the lethality associated with loss of *SLN1* in *S. cerevisiae* although *SLN1* is not an essential gene in *C. albicans* (Nagahashi et al., 1998). In addition, loss of *SLN1* causes constitutive activation of Hog1 in *C. albicans* as in *S. cerevisiae* (Roman et al., 2005). Collectively these observations indicate that *C. albicans* Sln1 likely functions as an osmosensor, however 2-component independent mechanisms also exist in the relay of osmotic stress signals to Hog1 (Chauhan et al., 2003). The *C. albicans* Nik1 histidine kinase is predicted to be cytoplasmic and, similar to other Nik1-related kinases, contains a number of HAMP (Histidine kinases, Adenylate cyclases, Methyl-accepting proteins, and Phosphatases) domains. Notably, efforts to generate a *nik1*Δ*/sln1*Δ double mutant proved unsuccessful (Yamada-Okabe et al., 1999), possibly implying functional redundancy between these kinases.

The function of Nik1 in Hog1 regulation is unclear. A recent high-throughput screen for pharmacologically active compounds that enhance the fungicidal activity of the cell wall targeting echinocandin, caspofungin, found that the metal chelator DTPA demonstrated the greatest synergistic activity by chelating magnesium (Polvi et al., 2016). Suppressor mutants were isolated that displayed resistance to the caspofungin and DTPA drug combination and remarkably, three of the four sequenced mutants were found to contain mutations in the *NIK1* gene. Such *NIK1* mutations impaired Hog1 activation in response to caspofungin and DTPA, which likely underlies the stress-resistant phenotype, as cells lacking Hog1 were also found to be resistant to this drug combination (Polvi et al., 2016). In a similar vein, the phenylpyrrole fungicide fludioxonil, used on crops worldwide, appears to exert its antifungal effects via activation of the Hog1 signaling pathway in a mechanism involving the Nik1 class of histidine kinases. For example, expression of *C. albicans NIK1* in *S. cerevisiae* renders this yeast hypersensitive to fludioxonil in a Hog1-dependent manner (Buschart et al., 2012). Furthermore, the HAMP domains within *C. albicans* Nik1 are important for the lethal activation of Hog1p in *S. cerevisiae* following fludioxonil treatment (Buschart et al., 2012). Thus, Nik1 appears to drive activation of Hog1 in response to distinct antifungals which promotes fungicidal activity.

The third histidine kinase in *C. albicans*, Chk1, is also predicted to be cytoplasmic and is a homolog of Mak2 and Mak3 in *Schizosaccharomyces pombe*. In *S. pombe*, Mak2 and Mak3 relay oxidative stress signals to the Sty1 SAPK module (Buck et al., 2001), but Chk1 is dispensable for hydrogen peroxide-induced activation of Hog1 in *C. albicans* (Li et al., 2004; Roman et al., 2005). However, *chk1*Δ cells show sensitivity to oxidative stress (Li et al., 2004), and using a LacZ reporter, it was found that transcription of *CHK1* was increased in response to oxidative stress (Li et al., 2004). Interestingly, Chk1 also appears to modulate the *C. albicans* cell wall (Li et al., 2004), but it is unknown if this is mediated via Hog1 regulation. Importantly, all three histidine kinases in *C. albicans* are needed for virulence (Calera et al., 1999; Yamada-Okabe et al., 1999; Selitrennikoff et al., 2001; Torosantucci et al., 2002), which due to their absence in metazoans makes such proteins potential antifungal candidates (Shor and Chauhan, 2015).

As in *S. cerevisiae, C. albicans* has a single phosphorelay protein which is homologous to *S. cerevisiae* Ypd1p. Although deletion of *YPD1* is lethal in *S. cerevisiae* (Maeda et al., 1994), due to the constitutive activation of Hog1 leading to apoptosis (Vendrell et al., 2011), this is not the case in *C. albicans*. Deletion of *YPD1* does trigger the constitutive activation of *C. albicans* Hog1 (Mavrianos et al., 2014; Day et al., 2017b), but cells adapt to this in the short term by inducing protein tyrosine phosphatase genes that prevent lethal levels of Hog1 phosphorylation (Day et al., 2017b). In addition, following long term sustained Hog1 phosphorylation, *C. albicans* mounts a distinct mechanism that reduces Hog1 phosphorylation such that *ypd1*Δ cells become phenotypically indistinguishable from wild-type cells (Day et al., 2017b). Interestingly, and perhaps surprisingly, down-regulation of *YPD1* expression in a murine model of systemic candidiasis actually enhanced the virulence of *C. albicans*. Whilst the mechanism underlying the enhanced virulence triggered by *YPD1* loss requires investigation, in contrast to previous suggestions (Shor and Chauhan, 2015), this indicates that Ypd1 is not a suitable antifungal drug target in *C. albicans* (Day et al., 2017b).

Downstream of Ypd1 is the Ssk1 response regulator. In *S. cerevisiae*, unphosphorylated, but not phosphorylated, Ssk1p drives the activation of the Ssk2p/Ssk22p MAPKKKs (Posas and Saito, 1998). Interestingly, in *C. albicans*, deletion of the analogous *SSK1* gene does not impact on Hog1 activation in response to osmotic stress. Instead, Ssk1 was shown to impact on Hog1 activation following oxidative stress, and consistent with this *ssk1*Δ cells display sensitivity to oxidative stress (Chauhan et al., 2003). However, it appears that none of the histidine kinases in *C. albicans* sense oxidative stress to regulate phosphorelay to Ssk1, as none impact on oxidative-stress induced activation of Hog1 (Roman et al., 2005). Thus, Ssk1 may relay oxidative stress signals to Hog1 in a mechanism independent of two-component signaling. Importantly, Ssk1 is essential for *C. albicans* virulence (Calera et al., 2000), and thus is potentially a more suitable antifungal target than Ypd1.

Two component independent mechanisms are also clearly involved in Hog1 activation in *C. albicans*. The redox-sensitive thioredoxin peroxidase, Tsa1, and the thioredoxin, Trx1, which reduces oxidized Tsa1, are required for H_2_O_2_-induced Hog1 activation (da Silva Dantas et al., 2010). However, Trx1 likely regulates Hog1 in a distinct mechanism than Tsa1, as the cysteine residues of Tsa1 regulated by Trx1 are not required for Hog1 activation (da Silva Dantas et al., 2010). In *S. pombe*, the Tsa1 ortholog, Tpx1, is similarly required for activation of the Sty1 SAPK in response to oxidative stress via a mechanism involving the formation of an intermolecular disulphide bond with Sty1 (Veal et al., 2004). However, *C. albicans* Hog1 is only oxidized following nitrosative stress and not oxidative stress (Herrero-de-Dios et al., 2018), suggesting that Tsa1 does not form a mixed disulphide with Hog1 following H_2_O_2_ stress. In addition to Tsa1/Trx1 in regulating H_2_O_2_-induced Hog1 activation, deletion of a mitochondrial biogenesis factor, Fzo1, also impairs oxidative stress-induced Hog1 phosphorylation (Thomas et al., 2013). Thus, mitochondrial function may also impact on oxidative stress induced activation of Hog1. *C. albicans* Hog1 is also negatively regulated by the Ptp2 and Ptp3, and Ptc1, phosphatases (Su et al., 2013; Sellam et al., 2019). Finally, although the pathways described above regulate Hog1 phosphorylation levels, a recent study revealed that oxidation of Hog1 is implicated in nitrosative-stress signaling (Herrero-de-Dios et al., 2018). However, the mechanism by which oxidation modulates Hog1 activity requires further investigation.

Regarding downstream responses regulated by Hog1, transcript profiling analysis revealed Hog1 to regulate the *C. albicans* transcriptome both in the absence and presence of stress (Enjalbert et al., 2006). Under basal conditions Hog1 represses subsets of stress-related genes and hyphae specific genes. The upregulation of hyphae specific genes is consistent with the filamentous phenotype of this mutant (Enjalbert et al., 2006), whereas the upregulation of genes involved in reactive oxygen species (ROS) metabolism may reflect the observation that *hog1*Δ cells have higher levels of intracellular ROS than wild-type cells (Alonso-Monge et al., 2009). Hog1 also plays a major role in the induction of core stress genes, which are commonly upregulated in response to osmotic, oxidative, and cadmium-imposed heavy metal stress, in addition to genes specifically induced in response to osmotic and heavy metal stress (Enjalbert et al., 2006). Intriguingly, a recent study revealed that in contrast to that reported in *S. cerevisiae* (Westfall et al., 2008), stress-induced gene expression in *C. albicans* can occur when the nuclear accumulation of Hog1 is prevented (Day et al., 2017a). Notably preventing Hog1 nuclear accumulation also had no impact on *C. albicans* virulence, suggesting that cytoplasmic functions of Hog1 may be more important (Day et al., 2017a). Furthermore, although Hog1 also plays a major role in the regulation of nitrosative stress genes, Hog1 is not noticeably phosphorylated following nitrosative stress (Herrero-de-Dios et al., 2018). Instead, Hog1 is oxidized following nitrosative stress suggesting that this post-translational modification can also modulate Hog1 function in *C. albicans* (Herrero-de-Dios et al., 2018). However, in contrast to the aforementioned stresses, Hog1 is largely dispensable for oxidative stress-induced gene expression (Enjalbert et al., 2006), even though *hog1*Δ cells are sensitive to ROS (Alonso-Monge et al., 2003). This suggests that Hog1 plays a non-transcriptional role in promoting oxidative stress tolerance in *C. albicans*. In this regard, it is noteworthy that Hog1 promotes the recovery from oxidative stress-induced cell cycle arrest (Correia et al., 2016). Furthermore, following oxidative stress the cell wall integrity MAPK Mkc1 is rapidly phosphorylated in a Hog1-dependent mechanism. However, this is unlikely to underlie the role of Hog1 in H_2_O_2_ resistance, as Mkc1 is dispensable for oxidative stress tolerance (Navarro-Garcia et al., 2005).

Regarding downstream targets of Hog1, two genes that encode putative substrates for the SAPK are transcriptionally induced following osmotic stress in a Hog1-dependent manner. The first is the Rck2 serine-threonine protein kinase, homologs of which are phosphorylated by the Hog1p and Sty1 SAPKs in the model yeasts *S. cerevisiae* and *S. pombe*, respectively (Bilsland-Marchesan et al., 2000; Smith et al., 2002). Interestingly deletion of *RCK2* in *C. albicans* renders cells sensitive to rapamycin, a phenotype shared by *hog1*Δ cells (Li et al., 2008). The second is Sko1, a CRE-binding transcriptional repressor that is a well-characterized Hog1p-target in *S. cerevisiae* (Proft and Struhl, 2002). Sko1 is phosphorylated by Hog1 (Rauceo et al., 2008) and this transcription factor regulates a subset of Hog1-dependent osmotic stress genes (Marotta et al., 2013). Moreover, Hog1 mediated regulation of Sko1 is linked to the filamentous phenotype exhibited by *hog1*Δ cells. Basal levels of Hog1 phosphorylation are needed to regulate Sko1 to dampen the expression of *BRG1*, a transcription factor required for the induction of hyphal genes (Su et al., 2013). In cells lacking Hog1, unphosphorylated Sko1 no longer binds to the *BRG1* promoter, which allows for *BRG1* expression and filamentation (Su et al., 2013). However, other transcriptional targets of Hog1p in *S. cerevisiae*, such as Msn2p and Msn4p, appear to be functionally reassigned in *C. albicans* (Nicholls et al., 2004). In a recent study, roles for Hog1 linking cell growth and division in *C. albicans* via transcription factor regulation have been uncovered (Sellam et al., 2019). Cells lacking Hog1 are significantly smaller than wild-type cells via a mechanism in which basal levels of Hog1 activation inhibit the SBF G1/S transcription factor complex thus delaying transition into S phase of the cell cycle. The Ptc1 and Ptc2 phosphatases, by modulating Hog1 phosphorylation, also govern the timing of Start. In parallel, Hog1 physically interacts with Sfp1, the transcription factor that regulates ribosome biogenesis, and recruits Sfp1 to ribosome biogenesis genes (Sellam et al., 2019). This interaction is abolished by stress providing a mechanism by which the timing of Start is regulated in part by modulation of the Hog1-Sfp1 interaction.

## *Aspergillus Fumigatus* SakA and MpkC

**Figure 3:**
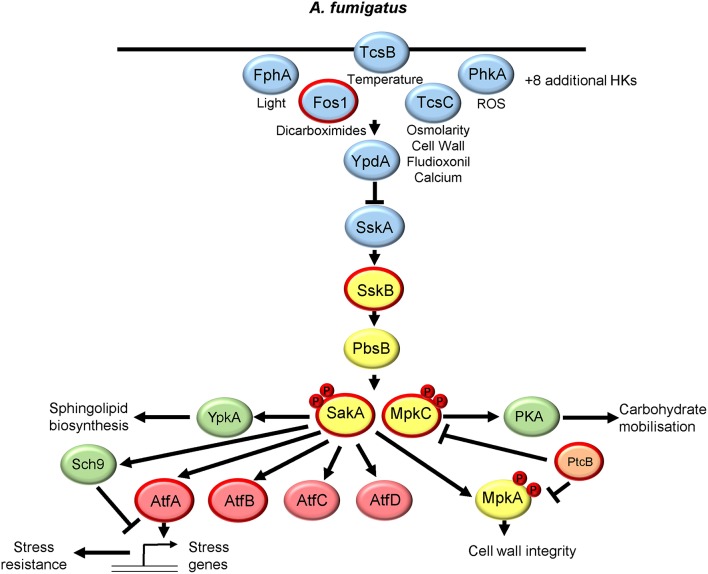
The SakA and MpkC SAPK pathway in *Aspergillus fumigatus*. Both the signaling proteins that regulate SakA and MpkC and the downstream responses are shown. See text for details. Those proteins circled in red are required for *A. fumigatus* virulence.

The genus *aspergillus* consists of several 100 species with *Aspergillus fumigatus* being the most important fungal pathogen of humans, causing ~65% of all invasive fungal infections in humans (Brown et al., 2012). Intriguingly, *A. fumigatus* contains two homologs of Hog1 known as SakA and MpkC, which share 68.4% identity. The role and regulation of the SakA and MpkC pathways is summarized in Figure 3. Analysis of single mutants in SakA revealed roles in adaptation to all main antifungal classes (polyenes, azoles, and echinocandins) and cold stress tolerance (Kim et al., 2012; Wong Sak Hoi et al., 2012; Altwasser et al., 2015), whereas MpkC function was linked to carbon source utilization (Reyes et al., 2006). Subsequently, a comparison of single and double mutants revealed that SakA and MpkC coordinate osmotic stress resistance with both displaying osmotic stress-induced nuclear accumulation albeit with different kinetics (Bruder Nascimento et al., 2016). However, in response to other stresses, SakA seems to be the most important, as Δ*sakA* and Δ*mpkC* Δ*sakA* mutants, but not the Δ*mpkC* strain, display notable increased sensitivity to ROS, cell wall damaging agents, and the cell wall-targeting antifungals caspofungin and nikkomycin Z (Bruder Nascimento et al., 2016). Furthermore, Δ*sakA and* Δ*mpkC* Δ*sakA* strains displayed alterations in cell wall composition and were less adherent than the wild-type strain (Bruder Nascimento et al., 2016). Such phenotypes may be linked to the interesting observation that activation of the MpkA cell wall integrity MAPK pathway in response to osmotic or cell wall stress was largely dependent on SakA and MpkC (Bruder Nascimento et al., 2016). Most importantly, SakA and MpkC likely play redundant roles in the virulence of this important fungal pathogen, as only the Δ*mpkC* Δ*sakA* strain and not the single mutants displayed highly attenuated virulence in a mouse neutropenic model of invasive pulmonary aspergillosis (Bruder Nascimento et al., 2016). Functioning upstream of Hog1 is a single MAPKK PbsB and, as in *C. albicans*, a single MAPKKK SskB. SakA phosphorylation is abolished in Δ*pbsB* cells (Ji et al., 2012) and Δ*sskB* cells (de Castro et al., 2014), and both mutants display acute sensitivity to cationic stress similar to that seen for Δ*sakA* mutants (de Castro et al., 2014). Furthermore, similar to cells lacking both SakA and MpkC, the Δ*sskB* mutant was found to display significantly attenuated virulence in an invasive pulmonary model of aspergillosis (de Castro et al., 2014) emphasizing the importance of SAPK signaling for *A. fumigatus* survival in the host.

Similar to that first reported in *S. cerevisiae*, in *A. fumigatus* two-component related phosphorelay systems likely act upstream of SskB to regulate SakA. However, filamentous fungi have more histidine kinases compared to yeasts, with the *A. fumigatus* genome containing 13 histidine kinases (Chapeland-Leclerc et al., 2015). The first to be characterized was Fos1/TcsA, which contains a PAS domain, with the Δ*fos1* strain displaying high levels of resistance to dicarboximide fungicides (Pott et al., 2000) and highly attenuated virulence in a murine model of systemic aspergillosis (Clemons et al., 2002). A homolog of Sln1, named TcsB, is also present and this is the only histidine kinase predicted to be located at the cell wall (Du et al., 2006), and regulates SakA in response to temperature stress (Ji et al., 2012). The histidine kinase associated with most phenotypes in *A. fumigatus* is a homolog of *C. albicans* Nik1, TcsC, which has six HAMP domains. Phenotypes linked to TcsC include reduction of conidiation, increased tolerance to cell wall-perturbing reagents and the fungicide fludioxonil, and enhanced sensitivity to osmotic stress stimuli and calcium stress (McCormick et al., 2012; Hagiwara et al., 2013; de Castro et al., 2014). Conflicting reports exist as to whether TcsC regulates SakA phosphorylation in response to such stresses (McCormick et al., 2012; Hagiwara et al., 2013), although the basal level of SakA phosphorylation is increased in Δ*tcsC* cells indicating that the stress-induced inactivation of TcsC triggers SakA phosphorylation (Hagiwara et al., 2013). *A. fumigatus* also has a homolog of the red light receptor histidine kinase, FphA, which in *A. nidulans* causes light dependent phosphorylation of SakA (Yu et al., 2016). Interestingly, FphA also regulates photoresponsive behaviors of *A. fumigatus* (Fuller et al., 2013), which raises the possibility that some of these responses may be mediated through SakA. Recently, a systematic analysis of 11 *A. fumigatus* histidine kinase gene deletions has been undertaken (Chapeland-Leclerc et al., 2015). Although most of the previously uncharacterized histidine kinase mutants failed to display strong phenotypes, cells lacking the class X histidine kinase PhkA displayed a clear hypersensitivity to oxidative stress agents (Chapeland-Leclerc et al., 2015). Notably, PhkA is homologous to the oxidative stress sensing Mak2 and Mak3 histidine kinases in *S. pombe* (Buck et al., 2001), although it remains to be tested whether PhkA relays oxidative stress signals to SakA.

Downstream of the histidine kinases, a homolog of the Ypd1 phosphorelay protein is present in the *A. fumigatus* genome but remains uncharacterized. However, a homolog of the Ssk1 response regulator SskA has been characterized, with Δ*sskA* cells displaying sensitivity to osmotic stress and no detectable phosphorylation of the SakA SAPK (Hagiwara et al., 2013). This implies that activation of Sak1 in response to osmotic stress is entirely dependent on the two-component related signaling system, in contrast to that seen in *C. albicans* in which osmotic stress signaling to the Hog1 SAPK occurs independently of Ssk1 (Chauhan et al., 2003).

Regarding two-component independent regulation of SakA/MpkC activation, the phosphatase PtcB, a homolog of the Ptc2p-Ptc3p type 2C serine/threonine phosphatases that dephosphorylate *S. cerevisiae* Hog1p, regulates SakA (Winkelstroter et al., 2015). Cells lacking PtcB display high basal levels of SakA phosphorylation and higher basal and stress-induction of the osmostress genes *catA, dprA*, and *dprB* (Winkelstroter et al., 2015). In addition, the Δ*ptcB* strain showed impaired virulence in a mouse model of invasive pulmonary aspergillosis (Winkelstroter et al., 2015), but whether this is due to SakA deregulation is unclear as PtcB also regulates the activity of the MpkA cell wall integrity MAPK pathway (Winkelstroter et al., 2015). Interestingly the calcium responsive CrzA transcription factor has been linked to SakA activation in *A. fumigatus*, as *phkB* histidine kinase and *sskB* MAPKKK gene expression require CrzA (de Castro et al., 2014). In addition, whilst basal levels of SakA phosphorylation are increased in Δ*crzA* cells, no further osmotic stress increase in SAPK phosphorylation is seen (de Castro et al., 2014).

Turning to the downstream responses elicited following SakA/MpkC activation, transcript profiling of single and double SAPK mutants revealed independent and overlapping roles for SakA and MpkC, with SakA playing a predominant role (Pereira Silva et al., 2017). Genes that require SakA for osmotic stress induction showed enrichment in the GO terms; cellular response to oxidative and osmotic stresses, trehalose catabolic process, and two-component signal transduction. In contrast, MpkC regulated genes did not display any significant GO term enrichment (Pereira Silva et al., 2017). However both SAPKs regulated the osmotic stress-repression of genes involved in cell cycle, metabolism and ribosome biogenesis (Pereira Silva et al., 2017). Interestingly, MpkC and SakA influence the expression of genes within the two-component related phosphorelay, and the SAPK module, which regulate their activation (Pereira Silva et al., 2017). In addition, a large number of kinases and transcription factors, some of which have previously been linked to SAPK function in *S. cerevisiae* or *S. pombe*, were found to be induced in a SAPK-dependent manner. One such kinase, SchA, is an ortholog of Sch9p, which is a chromatin-associated transcriptional activator of osmostress-responsive genes in *S. cerevisiae* (Pascual-Ahuir and Proft, 2007). However, in *A. fumigatus* cells lacking SchA, SakA phosphorylation, and SakA-dependent gene expression are sustained following osmotic stress, and cells lacking both Sch9 and SakA are much more sensitive to osmotic stress than the single mutants (de Castro et al., 2014). This suggests that SchA has a different role in mediating SAPK responses than in *S. cerevisiae*. In addition, the regulatory (PkaR) and catalytic (PkaC) subunits of protein kinase A were found to be induced in response to osmotic stress in a SakA and MpkA dependent manner (Pereira Silva et al., 2017). Subsequently, PKA activity was shown to be significantly reduced in Δ*sakA* and Δ*sakA* Δ*mpkC* strains and, intriguingly, co-immunoprecipitation experiments revealed that PKA and SakA physically interact (de Assis et al., 2018). Specifically, under non-stress conditions both regulatory and catalytic PKA subunits interact with SakA, but following osmotic stress the PkaR subunit dissociates, which is suggested to trigger PKA activity and trehalose and glycogen degradation (de Assis et al., 2018). Thus, SakA/MpkC have both transcriptional and posttranscriptional roles in regulating PKA activity in *A. fumigatus*. Interestingly, SakA has also recently been reported to physically interact with the AGC kinase YpkA which regulates sphingolipid synthesis (Fabri et al., 2018). Regarding potential downstream transcription factors, orthologs of the *S. pombe* SAPK substrate Atf1 (Lawrence et al., 2007), namely AtfA, B, C, and D, were induced in wild-type cells in a SakA dependent manner following osmotic stress (Pereira Silva et al., 2017). AtfA is the most important for stress tolerance in both *A. fumigatus* conidia (Hagiwara et al., 2014), and during mycelial growth (Pereira Silva et al., 2017). Furthermore, AtfA, and to a lesser extent AtfB, contribute to virulence (Pereira Silva et al., 2017). Whether such transcription factors are substrates for the SakA/MpkC SAPKs, as in *S. pombe*, remains to be tested. Moreover, as there are many additional transcription factors and kinases that are regulated by SakA/MpkC, much is still to be learnt about the SAPK-mediated regulatory networks in this important fungal pathogen of humans.

## *Cryptococcus Neoformans* Hog1

**Figure 4:**
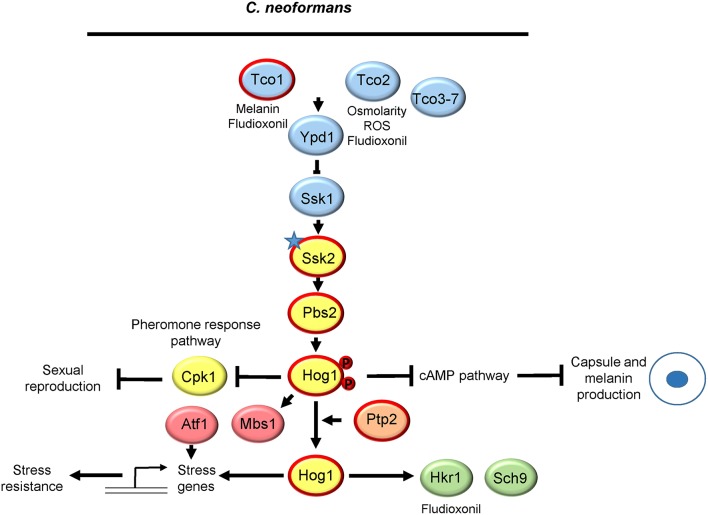
The Hog1 SAPK pathway in *Cryptococcus neoformans*. Both the signaling proteins that regulate Hog1 and the downstream responses are shown. The polymorphism in Ssk2 that drives Hog1 activation in the absence of stress is indicated with a star. See text for details. Those proteins circled in red are required for *C. neoformans* virulence.

There are seven species of *Cryptococcus*, but research into Hog1 signaling has thus far been restricted *to C. neoformans* (Serotype A) *and C. deneoformans* (Serotype D) (Hagen et al., 2015). *C. neoformans* (Serotype A) is the major species responsible for cryptococcal meningitis, resulting in over 180,000 deaths each year (Rajasingham et al., 2017). The role and regulation of the *C. neoformans* serotype A Hog1 pathway is summarized in Figure 4. The core SAPK signaling module is conserved comprising of the Hog1 SAPK, the Pbs2 MAPKK and, as in *C. albicans* and *A. fumigatus*, a single MAPKKK Ssk2 (Bahn et al., 2007). Intriguingly, in the majority of *C. neoformans* serotype A strains tested and in many, but not all, *C. deneoformans* serotype D strains, Hog1 regulation is unique in that Hog1 is constitutively phosphorylated under non-stress conditions and is actively dephosphorylated in response to various stress stimuli (Bahn et al., 2005). In contrast, in the serotype D strains that do not show high basal levels of Hog1 phosphorylation, typical stress-induced increases in Hog1 phosphorylation are seen. Notably, strains displaying high basal levels of Hog1 phosphorylation are generally more stress resistant. An elegant genetic approach identified polymorphisms in the *SSK2* gene as responsible for the variation in basal levels of Hog1 phosphorylation in different strain backgrounds, one of which (L240F) resides in the putative Ssk1 binding domain (Bahn et al., 2007). Interchange of *SSK2* alleles between strains showing either high or low Hog1 phosphorylation levels verified that polymorphisms in Ssk2 underlie the differential regulation of the Hog1 MAPK pathway (Bahn et al., 2007).

*Cryptococcus neoformans* Hog1 regulates a panoply of virulence attributes. With regard to stress resistance the Hog1 pathway promotes resistance to stresses likely encountered in the host and the environment, including osmotic and oxidative stresses, heat stress, UV irradiation, and HU and MMS-imposed genotoxic stresses, antifungal drugs including amphotericin and the toxic metabolic by-product methylglyoxal (Bahn et al., 2005, 2006, 2007; Lee et al., 2014a). It is intriguing that many such stress stimuli trigger the dephosphorylation of Hog1, indicating that stress-induced inactivation of Hog1 promotes stress resistance in *C. neoformans*. A common response to stress to conserve energy is to inhibit the energy demanding process of ribosome biogenesis, and in *C. neoformans* Hog1 function is important for the rapid degradation of ribosomal protein transcripts triggered by glucose starvation (Banerjee et al., 2016). Here it is postulated that ROS generated following glucose starvation may be the Hog1 regulatory signal (Banerjee et al., 2016). In contrast to processes requiring Hog1, cells lacking components of the Hog1 module are much more resistant to the antifungal agent fludioxonil (Bahn et al., 2005, 2007) which is consistent with the mode of action of fludioxonil mentioned previously, in stimulating the lethal hyperactivation of fungal SAPK pathways (Kojima et al., 2004). Interestingly, the high basal level of Hog1 phosphorylation seen in *C. neoformans* negatively impacts the production of two key virulence traits, capsule and melanin, likely via cross talk with the cAMP pathway (Bahn et al., 2005). Similarly, Hog1 also inhibits sexual reproduction via cross talk with the pheromone responsive Cpk1 MAPK pathway (Bahn et al., 2005). Consequently, *hog1*Δ, *pbs2*Δ, and *ssk2*Δ mutants all display enlarged capsule, increased melanin content, and enhanced mating proficiency (Bahn et al., 2005, 2007). Interestingly, despite the hyperproduction of capsule and melanin, deletion of core components of the MAPK module impairs *C. neoformans* virulence (Bahn et al., 2005, 2007).

As in other fungi, a two-component related phosphorelay system functions upstream of the SAPK module to relay environmental signals to *C. neoformans* Hog1 (Bahn, 2008). There are seven histidine kinases in *C. neoformans*, Tco1-7, which potentially facilitate sensing of multiple environmental cues (Bahn et al., 2006). Common to eukaryotic histidine kinases most contain a single histidine kinase and receiver domain although, intriguingly, Tco2 has two of each domain (Bahn et al., 2006). However, there is no ortholog of the Sln1 osmosensing histidine kinase, and none of the seven present histidine kinases possess a transmembrane domain suggesting that they sense intracellular stress signals (Bahn et al., 2006). Phenotypic analysis of Tco mutants identified Tco1 and Tco2 to have overlapping and specific functions in Hog1 regulation. Tco1 shows structural similarity to CaNik1 whereas Tco2 is unique. Loss of Tco2 resulted in impaired resistance to osmotic and oxidative stress and toxic metabolites, and the osmotic stress-induced decrease in Hog1 phosphorylation was delayed in *tco2*Δ cells. Both Tco1 and Tco2 contributed to fludioxonil sensitivity, with the double mutant displaying a similar level of resistance as *hog1*Δ cells and, consistent with the concept that Hog1 dephosphoryation accompanies stress resistance, Hog1 is trapped in its phosphorylated state in *tco1*Δ*tco2*Δ cells. Regarding the stress-independent phenotypes under Hog1 regulation, *tco1*Δ cells are mating defective but display enhanced melanin production similar to Hog1 pathway mutants (Bahn et al., 2006). Thus, Tco2 appears to play a key role in relaying stress signals to Hog1 whereas Tco1 is the key sensor kinase in regulating melanin synthesis via the SAPK module. In this regard it is interesting that Tco1 and not Tco2 is important for virulence (Bahn et al., 2006). The functions of the remaining Tco3-7 sensor kinases remain to be determined.

Homologs of the *S. cerevisiae* Ypd1p phosphorelay protein and Ssk1p response regulator are present in *C. neoformans*. Ssk1 functions as a global regulator of the Hog1 pathway (Bahn et al., 2006). The high basal levels of Hog1 phosphorylation are dependent on Ssk1, suggesting that the polymorphisms in Ssk2 that drive Hog1 phosphorylation do not hyperactivate the MAPKKK *per se*, but promote a positive interaction with Ssk1 that enhances Ssk2 activity. Moreover, *ssk1*Δ mutants display overlapping phenotypes with those in the core SAPK module components, including impacted stress resistance, increased capsule and melanin production, and enhanced mating (Bahn et al., 2006). The interesting exception to this is responses to osmotic stress, as osmotic stress-induced dephosphorylation of Hog1 and resistance to this stress are Ssk1-independent (Bahn et al., 2006). This suggests the existence of an additional two-component independent pathway that relays osmotic stress signals to Hog1 but, in contrast to *S. cerevisiae*, this is not governed by the Sho1/Msb2 pathway (So et al., 2018). As in *S. cerevisiae*, deletion of the Ypd1 phosphorelay protein gives a lethal phenotype in *C. neoformans* and this is dependent on Hog1 presence (Lee et al., 2011). This suggests that, as in other fungi, Ypd1 negatively regulates Hog1 and thus loss of Ypd1 drives lethal levels of Hog1 phosphorylation in *C. neoformans*.

Regarding negative regulators of Hog1 phosphorylation, transcript profiling experiments in *C. neoformans* revealed that Hog1 was required for both basal levels and the stress induction of the protein tyrosine phosphatases *PTP1* and *PTP2* (Ko et al., 2009). A subsequent phenotypic analysis revealed Ptp2 to be a major regulator of Hog1, and Ptp2 but not Ptp1 is important for virulence (Lee et al., 2014a). Disruption of *PTP2* increased the basal phosphorylation level of Hog1 and the stress induced dephosphorylation of Hog1 in response to osmotic stress was impaired in *ptp2*Δ cells. Cells lacking Ptp2 were actually more sensitive to osmotic stress than *hog1*Δ cells supporting the model that Ptp2-mediated dephosphorylation of Hog1 is needed to promote osmotic stress resistance (Lee et al., 2014a).

Concerning downstream responses of Hog1, transcript profiling analysis demonstrated that the SAPK module clearly regulates the *C. neoformans* transcriptome in the absence of stress with approximately 190 genes upregulated and 380 genes downregulated in *hog1*Δ cells compared to wild-type (Ko et al., 2009). Furthermore, a significant proportion of the deregulated genes were found to be Ssk1 dependent, consistent with previous work illustrating the importance of Ssk1 in Hog1 regulation (Bahn et al., 2006). Genes involved in melanin and capsule production, and those involved in the pheromone-Cpk1 MAPK pathway, were all upregulated in *hog1*Δ and *ssk1*Δ cells (Ko et al., 2009). This is highly consistent with the hyper-melanisation and encapsulation phenotypes, and enhanced mating exhibited by these mutants (Bahn et al., 2005, 2006). Interestingly, genes involved in ergosterol biosynthesis and resistance to heavy metals were upregulated in *hog1*Δ and *ssk1*Δ cells, promoting significantly higher ergosterol levels and cadmium resistance, respectively (Ko et al., 2009). The deregulation of ergosterol biosynthesis culminated with differential resistance profiles of Hog1 pathway mutants to the ergosterol-targeting antifungals, the triazoles and Amphotericin B; the increased induction of *ERG11* likely contributes to the resistance of *hog1*Δ mutants to Erg11-targeting triazoles, whereas enhanced sterol levels probably explains the heightened sensitivity of Hog1 pathway mutants to the ergosterol-binding drug Amphotericin B (Ko et al., 2009). With regard to stress-induced gene expression a large number of osmotic and oxidative stress responsive genes were found to be dependent on the SAPK module in line with the stress-sensitive phenotypes exhibited by Hog1 pathway mutants. Subsequently, a number of studies have been published describing the functional analysis of a number of Hog1 target genes including the cation transporters Ena1 and Nha1 (Jung et al., 2012), the aquaporin Aqp1 (Meyers et al., 2017), the sulfiredoxin gene Srx1 (Upadhya et al., 2013), and the ferroxidases Cfo1 and Cfo2 (Lee et al., 2014b). Some of these targets undoubtedly contribute to the stress protective roles of the Hog1 pathway. For example, Hog1 is absolutely essential for the oxidative stress mediated induction of *SRX1*, and *srx1*Δ mutants are highly sensitive to H_2_O_2_ as Srx1 functions to reduce the thioredoxin peroxidase Tsa1, which becomes hyper-oxidized (inactivated) during peroxide detoxification (Upadhya et al., 2013). In a similar vein, Hog1 controls basal expression and osmotic stress induction of the cation transporters Ena1 and Nha1, and phenotypic characterization of *ena1*Δ and *nha1*Δ mutants indicates this likely contributes to osmotic and cationic stress resistance especially in glucose limiting environments (Ko et al., 2009; Jung et al., 2012).

A number of regulatory proteins that potentially function downstream of Hog1 in *C. neoformans* have also been identified. These include the Hrk1 and Sch9 kinases and the Atf1 and Mbs1 transcription factors. Hrk1 is orthologous to the MAPK-activated protein kinases, Rck2 in *S. cerevisiae* (Bilsland-Marchesan et al., 2000) and Srk1 in *S. pombe* (Smith et al., 2002), which are substrates for the respective Hog1 and Sty1 SAPKs. In *C. neoformans HRK1* expression is Hog1 dependent and *hog1*Δ and *hrx1*Δ mutants display equivalent stress resistance to fludioxonil (Kim et al., 2011). Atf1 is orthologous to the *S. pombe* Atf1 transcription factor which is phosphorylated and regulated by the Sty1 SAPK (Lawrence et al., 2007). In *C. neoformans* Atf1 regulates some Hog1 dependent genes such as *PTP1* and *PTP3* (Lee et al., 2014a), and promotes resistance to several stresses (Missall and Lodge, 2005; Kim et al., 2010). However, whether Hrk1 or Atf1 is phosphorylated by Hog1 in *C. neoformans* is unknown, but the intriguing prediction based on stress-induced dephosphorylation of Hog1 is that phosphorylation of Hog1 substrates will be more dominant under non-stress conditions in this fungal pathogen.

## Concluding Remarks

Although SAPKs are among the most evolutionarily conserved stress-signaling proteins in fungi (Nikolaou et al., 2009), their role and regulation has significantly diverged amongst fungal species. This review has highlighted how, in addition to stress resistance, SAPKs co-ordinate a myriad of distinct responses, dependant on the fungal pathogen in question. Considering regulation, in contrast to the dogma that stress-induced phosphorylation promotes SAPK-mediated stress resistance, in many *C. neoformans* species the Hog1 SAPK is constitutively phosphorylated and stress-induced dephosphorylation promotes stress resistance and stress-induced gene expression. Moreover, nitrosative stress triggers oxidation rather than stress-induced phosphorylation of *C. albicans* Hog1. A further noteworthy difference is that *A. fumigatus* has two highly related SAPK proteins and both must be deleted to fully prevent SAPK-mediated responses. Whilst the core SAPK module and downstream transcriptional responses are fairly well-characterized in the fungal pathogens considered in this review, major gaps in our knowledge remain. For example little is known regarding; (i) the signals sensed by the majority of the histidine kinases in *C. albicans, A. fumigatus* and *C. neoformans*, (ii) the mechanisms underlying two-component independent signaling to SAPKs, (iii) the downstream substrates phosphorylated by the active SAPK, and (iv) the precise role of these signaling modules in promoting virulence. It is important that such questions are addressed as SAPK pathways are important virulence attributes in many human, plant and insect infecting fungal pathogens (Brown et al., 2017). Thus, the development of drugs which target fungal SAPKs has the exciting potential to generate broad-acting antifungal treatments.

## Author Contributions

JQ and AD contributed equally to the writing of this review.

### Conflict of Interest Statement

The authors declare that the research was conducted in the absence of any commercial or financial relationships that could be construed as a potential conflict of interest.
